# 144-week outcomes of lopinavir/ritonavir (LPV/r)-based first-line ART in 1,409 HIV-infected patients: data from the German STAR/STELLA cohort

**DOI:** 10.7448/IAS.17.4.19770

**Published:** 2014-11-02

**Authors:** Eva Wolf, Andreas Trein, Axel Baumgarten, Christoph Stephan, Hans Jaeger, Heribert Hillenbrand, Siegfried Koeppe, Thomas Lutz, Bettina Koenig, Hans-Juergen Stellbrink

**Affiliations:** 1 HIV Research and Clinical Care Centre, MVZ Karlsplatz, Munich, Germany; 2 HIV Center (Drs A. Schaffert/E. Schnaitmann/A. Trein), Stuttgart, Germany; 3 HIV Practice (Drs S. Dupke/A. Baumgarten/A. Carganico), Berlin, Germany; 4 HIV Center, University Hospital Frankfurt, Goethe University, Frankfurt, Germany; 5 MVZ PraxisCityOst (Drs. H. Hillenbrand, H. Karcher), Berlin, Germany; 6 Gemeinschaftspraxis (Drs S. Koeppe/P. Kreckel), Berlin, Germany; 7 Infektiologikum Frankfurt-City, Frankfurt am Main, Germany; 8 Medical Department, AbbVie Deutschland GmbH & Co. KG, Wiesbaden, Germany; 9 Study Center, Infektionsmedizinisches Centrum Hamburg, Hamburg, Germany

## Abstract

**Introduction:**

STAR/STELLA is a prospective[TS1] cohort of HIV patients initiated on LPV/r-based ART in routine clinical practice. Here, virologic/immunologic outcomes and safety data of LPV/r-based first-line ART over a period of 144 weeks are presented.

**Methods:**

Analysis included ART-naïve patients who started on LPV/r before July 2011 (i.e. patients with ≥144 weeks since ART initiation). Safety evaluation included adverse events (AEs), discontinuations (disc.) due to AEs, and symptoms assessed with the self-report ACTG Symptom Distress Module (ASDM; high score=high distress).

**Results:**

1409 patients were included (84% men; 76% on TDF+FTC), with a large proportion in advanced stages of HIV disease at ART initiation: 48% had a CD4 count <200/µL, 55% had HIV RNA levels >100,000 c/mL. 53% of patients (n=746) remained on LPV/r for at least 144 weeks. Time on drug was longer for patients initiated before 2008 than in subsequent years (HRadj, 1.2; 95% CI, 1.0–1.4; p=0.04; hazard ratio adjusted for CD4 <200/µL and HIV RNA >100,000 c/mL). Main reasons for d/c were: AEs (19.3%), patient wish (9.2%), virologic/immunologic failure (4.1%), and noncompliance (2.8%); 1.6% of patients died. By week 144, 33% of patients had >750 CD4/µL (Kaplan–Meier estimate): time to CD4 count >750 c/ µL, stratified by BL CD4 count, is shown in Figure 1.

ITT snapshot analysis of HIV RNA <50 c/mL at week 144 showed 51% responders (failure=d/c due to virologic/immunologic failure, AEs, noncompliance, death). In patients on LPV/r for 144 weeks, median CD4 change was +314/µL (IQR, 205–440/µL), 87% had HIV RNA levels <50 c/mL. In patients who discontinued therapy prior to week 144, 56% had an HIV RNA level <50 c/mL. In 51% of patients, ≥1 AE was reported (most commonly diarrhoea, 35%); 11% of patients had ≥1 AE of grade 3 or 4 (diarrhoea, 4.5%). In patients who remained on LPV/r based ART through 144 weeks, median ASDM score decreased significantly from 9 at BL (IQR, 3–21) to 2.5 at Week 144 (IQR, 0–8.5, p<0.001).

**Conclusion:**

In the STAR/STELLA observational cohort, LPV/r-based ART demonstrated good virologic outcomes and immune recovery in ART-naïve patients over 144 weeks, with significant improvements in symptom distress. Over three years, <5% of patients discontinued LPV/r due to virologic/immunologic failure, and 19% of patients discontinued for tolerability reasons.

**Figure 1:**
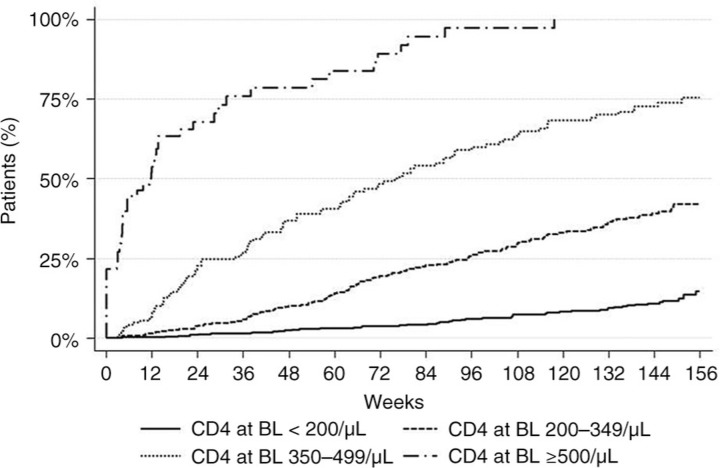
Time to CD4 count >750 c/μL, stratified by BL CD4 count.

